# Interplay among Antioxidant System, Hormone Profile and Carbohydrate Metabolism during Bud Dormancy Breaking in a High-Chill Peach Variety

**DOI:** 10.3390/antiox10040560

**Published:** 2021-04-04

**Authors:** José A. Hernández, Pedro Díaz-Vivancos, José Ramón Acosta-Motos, Nuria Alburquerque, Domingo Martínez, Esther Carrera, Jesús García-Bruntón, Gregorio Barba-Espín

**Affiliations:** 1 Group of Fruit Tree Biotecnology, CEBAS-CSIC, 30100 Murcia, Spain; pdv@cebas.csic.es (P.D.-V.); jacosta@cebas.csic.es (J.R.A.-M.); nalbur@cebas.csic.es (N.A.); gbespin@cebas.csic.es (G.B.-E.); 2 Department of Food Technology, University Miguel Hernandez, 03202 Orihuela, Spain; dmromero@umh.es; 3 Group of Hormonal Metabolism and Plant Development Regulation, IBMCP-CSIC, 46011 Valencia, Spain; ecarrera@ibmcp.upv.csic.es; 4 Group of Fruitculture, IMIDA, 30012 Murcia, Spain; jesus.garcia2@carm.es

**Keywords:** abscisic acid, antioxidant enzymes, chill requirement, dormancy, flower buds, gibberellins, peach, sugars

## Abstract

(1) Background: *Prunus* species have the ability to suspend (induce dormancy) and restart growth, in an intricate process in which environmental and physiological factors interact. (2) Methods: In this work, we studied the evolution of sugars, antioxidant metabolism, and abscisic acid (ABA) and gibberellins (GAs) levels during bud dormancy evolution in a high-chill peach variety, grown for two seasons in two different geographical areas with different annual media temperature, a cold (CA) and a temperate area (TA). (3) Results: In both areas, starch content reached a peak at ecodormancy, and then decreased at dormancy release (DR). Sorbitol and sucrose declined at DR, mainly in the CA. In contrast, glucose and fructose levels progressively rose until DR. A decline in ascorbate peroxidase, dehydroascorbate reductase, superoxide dismutase and catalase activities occurred in both seasons at DR. Moreover, the H_2_O_2_-sensitive SOD isoenzymes, Fe-SOD and Cu,Zn-SOD, and two novel peroxidase isoenzymes, were detected. Overall, these results suggest the occurrence of a controlled oxidative stress during DR. GA_7_ was the major bioactive GA in both areas, the evolution of its levels being different between seasons and areas. In contrast, ABA content decreased during the dormancy period in both areas, resulting in a reduction in the ABA/total GAs ratio, being more evident in the CA. (4) Conclusion: A possible interaction sugars-hormones-ROS could take place in high-chill peach buds, favoring the DR process, suggesting that, in addition to sugar metabolism, redox interactions can govern bud DR, regardless of chilling requirements.

## 1. Introduction

Deciduous woody plants, as stone fruit trees, are characterized by their capacity to interrupt and restart growth regularly in response to environmental and seasonal challenges [1]. In tree physiology, dormancy has been defined as the absence of visible growth in any plant structure containing a meristem. However, other authors proposed a definition of dormancy similar to that proposed for seed dormancy as “the incapability to achieve any growth from meristems under favorable conditions” [1]. Bud dormancy has typically been divided into three main phases: paradormancy (PD, controlled by other parts of the plant), endodormancy (ED, mainly controlled by internal factors of the flower bud) and ecodormancy (EC, controlled by external factors) [2]. A differential characteristic of bud dormancy is its quantitative nature, in relation to the chilling requirements for bud dormancy breaking [1]. This is the case of stone fruit trees. However, dormancy in not just a survival strategy, since cold temperatures during winter are also required for a proper flowering [3]. Plant hormones play crucial roles in bud dormancy regulation [4,5]. Particularly, abscisic acid (ABA) and bioactive gibberellins (GAs) are known to play antagonistic roles in bud dormancy, a high GAs content being related with dormancy release (DR) and high ABA content with the depth of dormancy [6]. In this sense, at the transcriptional level, changes in the expression of ABA-signaling and GAs-related genes have been observed as DR progresses [7]. Moreover, it is still unclear whether GA biosynthesis is induced during chilling phase or after DR [6]. While the role of ABA in seed dormancy has been well established, its function in bud dormancy remains to be elucidated [8]. Bud and seed dormancies in perennial plants have been shown to share common mechanisms. In this regard, diverse physiological and molecular studies have contributed to the elucidation of the involvement of ABA in bud dormancy [1,4,9,10].

Besides plant hormones, sugars may also act in the regulation of bud dormancy [4]. Soluble sugars are involved in the regulation of different physiological functions, including plant growth and development, energy source, synthesis of metabolites, and short- and long-distance signaling. In addition, sugars also act as osmoprotectant molecules as well as reactive oxygen species (ROS)-scavengers [11]. Transcriptomic and biochemical data indicated that sugar metabolism and signaling are involved in bud dormancy development [12]. In addition, a cross-talk between sugars and plant hormones during flowering induction has been reported [12,13]. This signaling network involves many pathways and contributes to orchestrate the transition among the different dormancy phases. Chilling temperatures affect carbohydrate metabolism in both flower buds and underlying tissues, although the response may be different depending on the plant tissue and species [3,14,15]. Short days and low temperatures in autumn activate starch accumulation in stems and buds. Stored starch can be degraded to soluble sugars in response to freezing temperatures during winter [12,16]. The accumulation of starch in plant species with high chill requirements reflects a good synchronization with the season providing a key adaptive advantage, and plays a clear role in flower development and in the reproductive process [3]. In the bark tissue of peach, plum and apricot stems, decreased starch content correlated with increased glucose and fructose levels and chill accumulation [14]. Similarly, in *Prunus mume*, bud starch and amylopectin levels were high at the beginning of dormancy and gradually declined until DR, showing soluble sugars, sucrose and glucose the opposite dynamic [12].

Oxidative stress appears to not only be a stress response during bud dormancy, but also an important signaling mechanism [17]. Likewise, ROS production and antioxidant system seem to be intimately linked factors in DR [18]. Sub-lethal ROS accumulation, especially H_2_O_2_, may activate bud DR [18,19,20,21,22]. During ED, H_2_O_2_ has been identified as a signal involved in antioxidant defenses induction, such as peroxidase (*POX*) and superoxide dismutase (*SOD*) genes [10,21]. It has been suggested that POX and plasma membrane-bound NADPH oxidases play an important role in the control of the redox environment regulating the quiescence to proliferation transition [23]. Recently, Conrad et al. (2020) reported 63 differentially expressed stress-related genes during the transition of ED to EC in apricot and peach, including some oxidative stress-related genes [24].

Upon a broad comparison of 204 peach cultivars, remarkable differences on chill requirements were found [25]. However, the physiological determinants of such differences remain to be elucidated. Herein, establishing the interplay between chilling requirements and possible physiological dormancy markers would allow a more accurate prediction of the dormancy evolution of a given cultivar.

The present study contributes to the understanding of the physiological and biochemical mechanisms involved in bud dormancy evolution in a high-chill peach variety. In this sense, we studied the interaction between antioxidant and sugar metabolisms, as well as the evolution in ABA and GAs contents in flower buds for two consecutive seasons (2017–2018; 2018–2019) in two geographical areas with different annual media temperature. Finally, the results of this work are compared with those recently published in our research group on a low-chill peach cultivar [26], leading us to stablish common determinants of peach bud dormancy maintenance and release.

## 2. Materials and Methods

### 2.1. Plant Material and Flower Bud Sampling

Floral buds from ‘Yumyeong’ (GEM020), a peach (*Prunus persica* L.) tree variety with high chill requirements (above 40 chill portions), were used. Plants were located in the experimental facilities of IMIDA Germplasm Bank (BAGERIM) in two different climatic locations in southeastern Spain: a temperate area (TA; El Jimenado, Torre Pacheco, Murcia 37°46′13.6″ N 1°01′16″ W) and other localization displaying colder temperatures (cold area, CA), especially in winter (El Chaparral, Bullas, Murcia 38°06′36.0″ N 1°41′19.8″ W). The Supplementary file 1 shows the dates for flower bud sampling conducted for the two growing seasons (from October 2017 to March 2018, and from October 2018 to March 2019), and their correspondence with the different dormancy stages, according to the Dynamic Model [27,28], using chill portions (CP) for chilling accumulation determination [29]. This model predicts the accumulation of an intermediate product triggered by cold temperatures, which may be reversed by warm temperatures. Once this intermediate product reached a certain level, CP are fixed and are not further influenced by warm temperatures [29]. In the CA the mean, minimum and maximum temperature registered were 13.8, 5.3 and 23.0 °C, respectively, for the first season, whereas the corresponding temperatures for the second season were 8.6, 1.5 and 17.2 °C. In the TA, the mean, minimum and maximum temperature were 16.7, 10.8 and 23.4 °C, respectively, for the first season, and 12.7, 7.9 and 18.5 °C for the second season. In every sampling time point, over 200 flower buds per tree were collected. Then, the flower buds were manually devoided of their bracts, and either directly used for histological study or maintained at −80 °C for further analysis.

### 2.2. Histological Study, Sugars Determination, and Enzymatic Antioxidants and Plant Hormones Analysis

The histological studies of flower buds, their starch and sugar contents as well as the determination of enzymatic antioxidants and plant hormones levels were performed as recently described in our research group [26]. Briefly, for histological studies 10 flower buds from each sampling point date were used.

Determination of enzymatic antioxidants was performed using approximately 0.25 g of flower buds. The extraction was performed according to [26]. In brief, buds were ground in liquid nitrogen, and extracted with 50 mm Tris-acetate buffer, pH 6.0 (1/8 *w*/*v*), containing 2 mM Cys, 0.1 mM EDTA, 1% PVP (*w*/*v*), 2% PVPP (*w*/*v*) and 0.2% Triton X–100 (*w*/*v*). After centrifugation at 10,000× *g* for 15 min at 4 °C, the resulting supernatant was filtered on Sephadex G-25 NAP columns (ThermoFisher Scientific, Hampton, NH, USA) equilibrated with the same buffer used for homogenization. Ascorbate-glutathione (ASC-GSH) cycle enzymes (ascorbate peroxidase, APX; dehydroascorbate reductase, DHAR; glutathione reductase, GR; monodehydroascorbate reductase, MDHAR), peroxidase (POX), catalase (CAT), and superoxide dismutase (SOD) activities were measured as described [30,31,32] using a UV/Vis V-630 Bio spectrophotometer (Jasco, Tokyo, Japan). In addition, non-denaturing electrophoretic analysis (PAGE) was performed to study the POX and SOD isoenzymes pattern [26].

Plant hormone analysis was performed using 0.1 g of flower buds, as reported [26]. In brief, ground buds in liquid nitrogen were homogenized in 80% methanol −1% acetic acid containing deuterium labelled gibberellins (GAs) and abscisic acid (ABA) as internal standards, and subjected to gentle agitation for one hour at 4°C. The resulting extract was maintained at 20 °C overnight and then centrifuged, and the supernatant was dried in a vacuum evaporator. The dry residue was suspended in 1% acetic acid and filtered through an Oasis HLB (reverse phase) column (Waters Corp., Milford, MA, USA). at Plant Hormones Quantification platform (IBMCP, Valencia, Spain) by Ultra Performance Liquid Chromatography-Mass Spectrometry (UPLC-MS), using a Thermo Scientific™ Q Exactive™ Hybrid Quadrupole-Orbitrap Mass Spectrometer. Further detail for hormones extraction and quantification are described in [26].

### 2.3. Statistical Analysis

The data were analyzed by one-way ANOVA using the SPSS 20.0 software (SPSS Inc., 2002) software. The treatment means were separated by Tukey’s Multiple Range Test (*p* ≤ 0.05). A principal component analysis (PCA) and partial least squares analysis for dimension reduction were done to assign the principal components having eigenvalues ≥ 1.0, using the StatGraphics Centurion XV software (StatPoint Technologies, Warrenton, VA, USA).

## 3. Results

### 3.1. Histological Analysis

**Figure 1:**
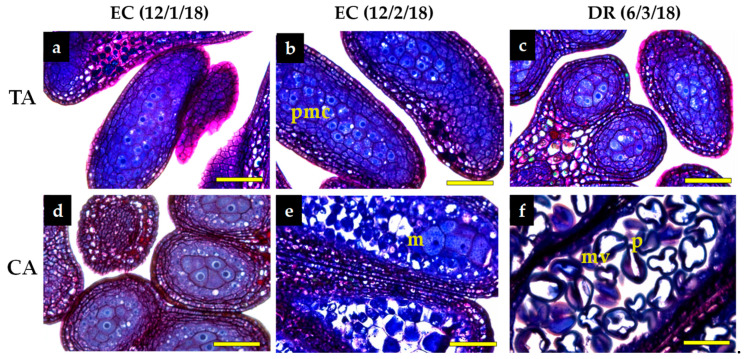
Histological analysis of flower bud evolution from peach cultivar ‘GEM020’ Samples were collected from a temperate (TA) (**a**–**c**) and a cold area (CA) (**d**–**f**) during ecodormancy (EC) (**a**,**b**,**d**,**e**) and dormancy release (DR) (**c**,**f**) m = microespore; vm = vacuolated microspore; pmc= pollen mother cell; *p* = pollen grain. Bars’ length corresponds to 50 µm.

The pollen developmental stage of the anthers was monitored in both growing seasons and areas, from the end of ED to DR. This is shown in Figure 1 for samples from the first season, as representative. At the end of ED, there was no evidence of pollen development in the anthers, the pollen being only present in mother cells (Figure 1a,d). By mid-EC, the pollen mother cells were still present in the TA (Figure 1b), whereas the anthers in the CA were in a more advanced developmental stage, showing isolated microspores after division of pollen mother cells (Figure 1c). At DR, clear differences on the developmental stage were observed between the two locations: anthers contained pollen mother cells in the TA (Figure 1c), whereas vacuolated microspores and pollen grains were observed in the CA (Figure 1f).

### 3.2. Carbohydrate Metabolism

**Figure 2:**
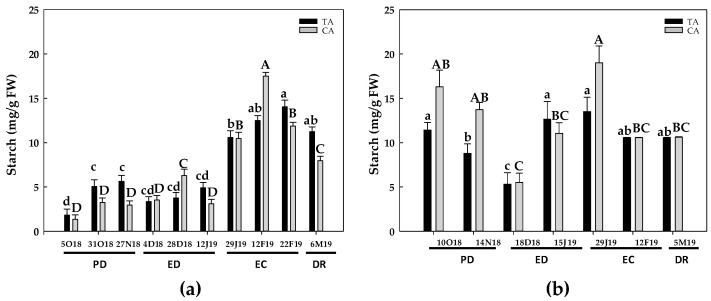
Evolution of starch contents in flower buds from peach ‘GEM020’ cultivated in two different geographical areas during the dormancy cycle on two different seasons. (**a**): season 1 (2017–2018); (**b**): season 2 (2018–2019). Data represent the mean ± SE, *n* = 5. Different letters (lowercase and uppercase for the samples of the temperate (TA) and the cold area (CA), respectively) indicate significant differences according to the Tukey’s multiple test (*p* ≤ 0.05). PD: paradormancy; ED: endodormancy; EC: ecodormancy; DR: dormancy release. FW: fresh weight. The chill units accumulated at the different times and locations are shown in Supplemental file 1.

The starch content of flower buds varied mainly in function of the growing season, whereas the differences between climatic locations were less evident (Figure 2).

In the season 2017–2018, in the TA, starch contents increased about 3 times at the end of PD, remaining constant until the end of ED. Then, starch contents strongly increased at EC, showing a peak at the end of EC (Figure 2a). Finally, starch contents slightly decreased at DR, although the levels remained about 6-times higher than the initial values (Figure 2a). In the CA, the behavior was somewhat similar, the starch contents showing a 5-fold increase at ED and peaking at EC, with a near 13-fold the initial values. Finally, starch content decreased at the end of EC and at DR, its levels at this point being, however, near 6-fold the initial values (Figure 2a).

In the second season (2018–2019), the initial starch values were much higher in both locations, in relation to the values observed for the first season. In general, starch decreased from PD to the initial ED, and then it increased at the end of ED, peaked at EC in the cold area, and remained constant in the temperate zone (Figure 2b). Finally, starch levels decreased in both locations at DR. In addition, in both seasons the highest mean starch levels were observed in the CA at EC (Figure 2).

**Figure 3:**
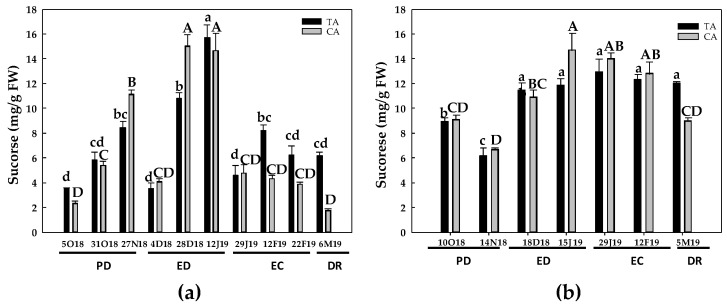
Evolution of sucrose contents in flower buds of peach ‘GEM020’ cultivated in two different geographical areas during the dormancy cycle on two seasons. (**a**): season 1 (2017–2018); (**b**): season 2 (2018–2019). Data represent the mean ± SE, *n* = 5. Different letters (lowercase and uppercase for the samples of the temperate (TA) and the cold area (CA), respectively) indicate significant differences according to Tukey’s multiple test (*p* ≤ 0.05). PD: paradormancy; ED: endodormancy; EC: ecodormancy; DR: dormancy release. FW: fresh weight. The chill units accumulated at the different times and locations are shown in Supplemental file 1.

**Figure 4:**
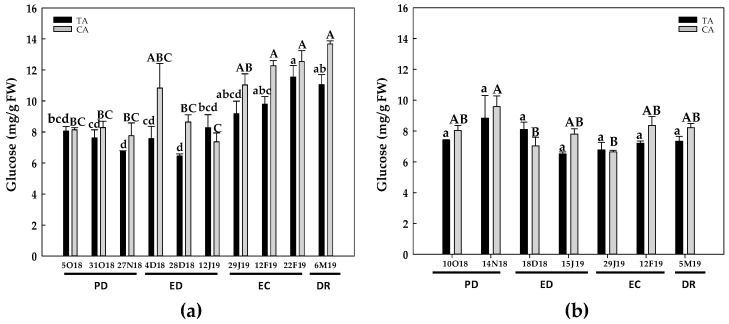
Evolution of glucose contents in flower buds of peach ‘GEM020’ cultivated in two different geographical areas during the dormancy cycle on two seasons. (**a**): season 1 (2017–2018); (**b**): season 2 (2018–2019). Data represent the mean ± SE, *n* = 5. Different letters (lowercase and uppercase for the samples of the temperate (TA) and the cold area (CA), respectively) indicate significant differences according to Tukey’s multiple test (*p* ≤ 0.05). PD: paradormancy; ED: endodormancy; EC: ecodormancy; DR: dormancy release. FW: fresh weight. The chill units accumulated at the different times and locations are shown in Supplemental file 1.

**Figure 5:**
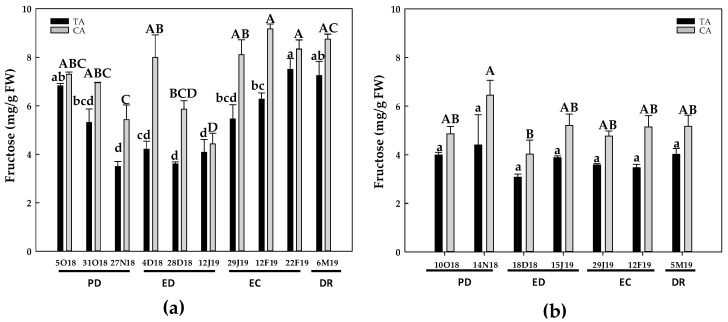
Evolution of fructose contents in flower buds of peach ‘GEM020’ cultivated in two different geographical areas during the dormancy cycle on two seasons. (**a**): season 1 (2017–2018); (**b**): season 2 (2018–2019). Data represent the mean ± SE, *n* = 3. Different letters (lowercase and uppercase for the samples of the temperate (TA) and the cold area (CA), respectively) indicate significant differences according to Tukey’s multiple test (*p* ≤ 0.05). PD: paradormancy; ED: endodormancy; EC: ecodormancy; DR: dormancy release. FW: fresh weight. The chill units accumulated at the different times and locations are shown in Supplemental file 1.

**Figure 6:**
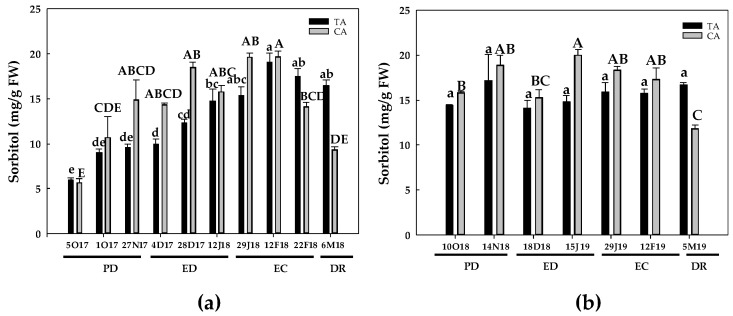
Evolution of sorbitol contents in flower buds of peach ‘GEM020’ cultivated in two different geographical areas during the dormancy cycle on two seasons. (**a**): season 1 (2017–2018); (**b**): season 2 (2018–2019). Data represent the mean ± SE, *n* = 5. Different letters (lowercase and uppercase for the samples of the temperate (TA) and the cold area (CA), respectively) indicate significant differences according to Tukey’s multiple test (*p* ≤ 0.05). PD: paradormancy; ED: endodormancy; EC: ecodormancy; DR: dormancy release. FW: fresh weight. The chill units accumulated at the different times and locations are shown in Supplemental file 1.

The evolution of soluble sugars contents was also somewhat different between the two experimental seasons (Figure 3, Figure 4, Figure 5 and Figure 6). However, sucrose (Figure 3) and sorbitol (Figure 6) and were the most abundant sugars in GEM020 peach buds for both seasons and locations.

The sucrose contents in the first season (Figure 3) increased at PD in both locations, especially in the cold area (5 and 2.3 times in the CA and TA, respectively), followed by a decrease at the beginning of ED in both regions, reaching the initial values. Then, sucrose contents peaked at the end of ED in both regions. Finally, sucrose declined at EC and DR, mainly in the CA (Figure 3a). In the second season, sucrose decreased at PD, the decline being statistically significant only in the TA (30% decrease). Again, sucrose levels peaked at ED in both cases, especially in the CA (60% increase vs. 30% increase in the TA). Finally, sucrose remained constant until DR in the TA, but decreased in the CA in relation to the values found at EC (Figure 3b).

During the first season and in both zones, the concentration of glucose remained statistically unchanged at PD and ED. Subsequently, a 40% increase at the end of EC and at DR was found in the TA in relation to the initial values. A similar response occurred in the CA. Glucose contents also remained constant at PD and ED, and a 50% increase took place at the end of EC and at DR, (Figure 4a). During the second season, glucose contents remained unchanged in all the dormancy period in the TA. In contrast, in the CA, glucose levels peaked at end of PD, followed by a 26% decrease at the beginning of ED and at EC. Finally, glucose levels increased at DR, reaching the initial values (Figure 4b).

During the first season, the levels of fructose in peach buds from the TA declined about 50% at the end of PD. Then, the fructose levels remained constant at ED, followed by consecutive increases at EC and DR took, reaching the initial values. In the CA, fructose increased from the end of PD to the beginning of ED, followed by a 40% decrease at the end of ED, and finally increased at EC and DR (Figure 5a). In contrast, during the second season, fructose levels remained unchanged in the TA. In the CA, fructose levels also remained statistically unalterable except for a slight decrease recorded from the end of PD to the beginning of ED (Figure 5b).

Regarding sorbitol, its contents remained statistically unchanged at PD in the TA during the first season, but a 60–70% increase was produced at ED. Then, sorbitol peaked at EC and remained unchanged until DR, although sorbitol levels were 2.8-fold higher in relation to its initial values (Figure 6a). In the CA, sorbitol increased during PD, reaching a 2.6-fold increase with respect to the initial values. The values remained constant at ED and at the onset of EC. Then, sorbitol concentration declined during EC and DR until the initial values were reached (Figure 6a). During the second season, no changes in sorbitol levels were noticed in the TA. In the CA, sorbitol remained constant at PD and at the onset of EC. Then, sorbitol showed a 27% increase at the end of ED and remained statistically unchanged until EC. Finally, a 25% decrease at DR in relation to the initial values was observed (Figure 6b).

In summary, starch content peaked at EC stage, and then decreased at DR in both locations. In GEM020 buds, sorbitol and sucrose were the most abundant soluble sugars, although their levels declined at DR. On the other hand, glucose and fructose levels progressively rose, reaching their highest values at DR.

### 3.3. Antioxidant Metabolism

**Figure 7:**
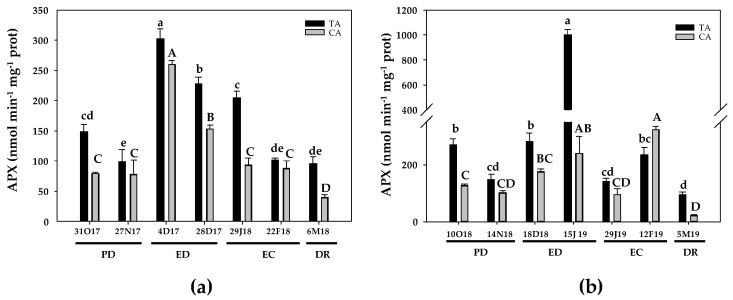
Evolution of ascorbate peroxidase (APX) activity in flower buds of peach ‘GEM020’ cultivated in two different geographical areas during the dormancy cycle on two seasons. (**a**): season 1 (2017–2018); (**b**): season 2 (2018–2019). Data represent the mean ± SE, *n* = 5. Different letters (lowercase and uppercase for the samples of the temperate (TA) and the cold area (CA), respectively) indicate significant differences according to Tukey’s multiple test (*p* ≤ 0.05). PD: paradormancy; ED: endodormancy; EC: ecodormancy; DR: dormancy release. FW: fresh weight. The chill units accumulated at the different times and locations are shown in Supplemental file 1.

**Figure 8:**
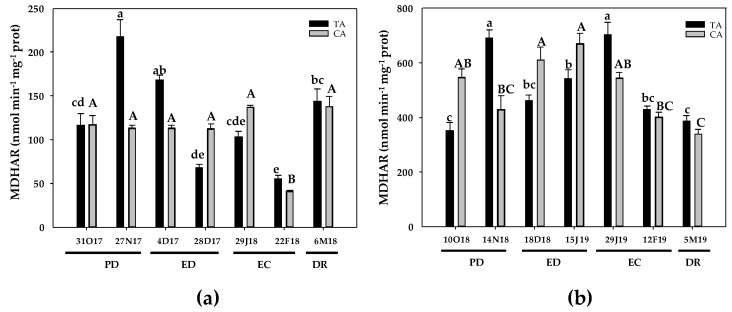
Evolution of monodehydroascorbate reductase (MDHAR) activity in flower buds of peach ‘GEM020’ cultivated in two different geographical areas during the dormancy cycle on two seasons. (**a**): season 1 (2017–2018); (**b**): season 2 (2018–2019). Data represent the mean ± SE, *n* = 5. Different letters (lowercase and uppercase for the samples of the temperate (TA) and the cold area (CA), respectively) indicate significant differences according to Tukey’s multiple test (*p* ≤ 0.05). PD: paradormancy; ED: endodormancy; EC: ecodormancy; DR: dormancy release. FW: fresh weight. The chill units accumulated at the different times and locations are shown in Supplemental file 1.

**Figure 9:**
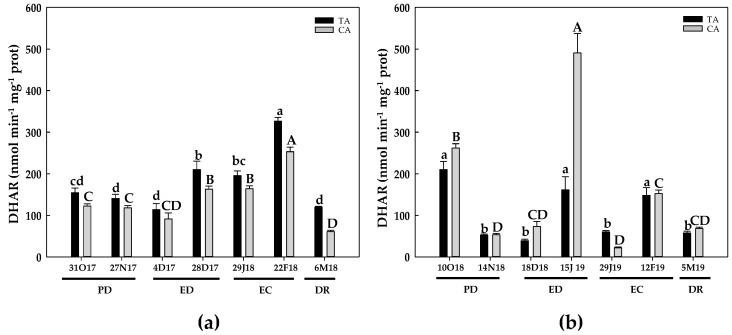
Evolution of dehydroascorbate reductase (DHAR) activity in flower buds of peach ‘GEM020’ cultivated in two different geographical areas during the dormancy cycle on two seasons. (**a**): season 1 (2017–2018); (**b**): season 2 (2018–2019). Data represent the mean ± SE, *n* = 5. Different letters (lowercase and uppercase for the samples of the temperate (TA) and the cold area (CA), respectively) indicate significant differences according to Tukey’s multiple test (*p* ≤ 0.05). PD: paradormancy; ED: endodormancy; EC: ecodormancy; DR: dormancy release. FW: fresh weight. The chill units accumulated at the different times and locations are shown in Supplemental file 1.

**Figure 10:**
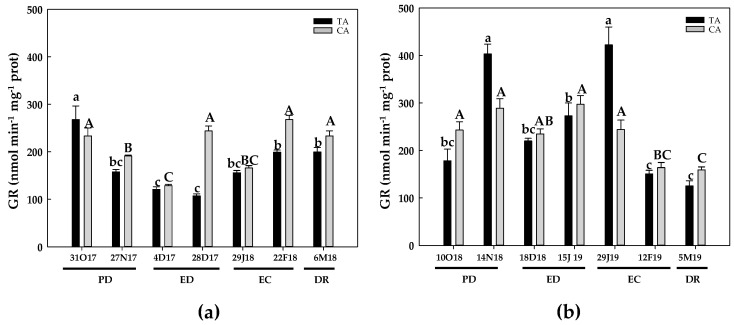
Evolution of glutathione reductase (GR) activity in flower buds of peach ‘GEM020’ cultivated in two different geographical areas during the dormancy cycle on two seasons. (**a**): season 1 (2017–2018); (**b**): season 2 (2018–2019). Data represent the mean ± SE, *n* = 5. Different letters (lowercase and uppercase for the samples of the temperate (TA) and the cold area (CA), respectively) indicate significant differences according to Tukey’s multiple test (*p* ≤ 0.05). PD: paradormancy; ED: endodormancy; EC: ecodormancy; DR: dormancy release. FW: fresh weight. The chill units accumulated at the different times and locations are shown in Supplemental file 1.

**Figure 11:**
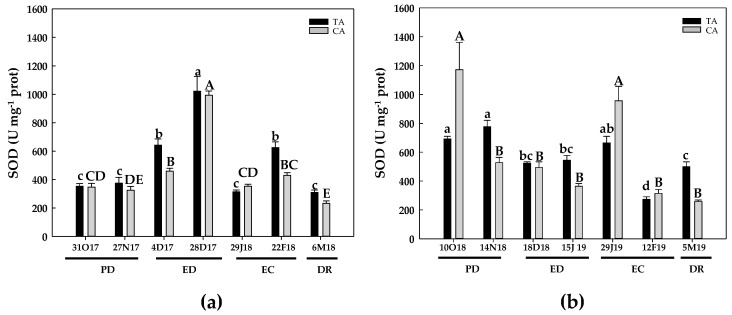
Evolution of superoxide dismutase (SOD) activity in flower buds of peach ‘GEM020’ cultivated in two different geographical areas during the dormancy cycle on two seasons. (**a**): season 1 (2017–2018); (**b**): season 2 (2018–2019). Data represent the mean ± SE, *n* = 5. Different letters (lowercase and uppercase for the samples of the temperate (TA) and the cold area (CA), respectively) indicate significant differences according to Tukey’s multiple test (*p* ≤ 0.05). PD: paradormancy; ED: endodormancy; EC: ecodormancy; DR: dormancy release. FW: fresh weight. The chill units accumulated at the different times and locations are shown in Supplemental file 1.

The activity of the ASC-GSH cycle enzymes as well of SOD, POX and CAT were studied in the floral peach buds (Figure 7, Figure 8, Figure 9, Figure 10 and Figure 11). As a part of the ASC-GSH cycle, APX enzymes function as high-affinity H_2_O_2_ scavengers at the expense of ascorbate. During the first season, in the TA, APX activity decreased about 32% at PD, then increased 2-fold at the onset of ED, followed by a 53% increase at the end of this period and a 38% increase at the onset of EC. Finally, a decrease at the end of EC and at DR took place, reaching the initial values (Figure 7a). In the CA, APX activity remained constant at PD and strongly enhanced at the onset of ED (3-fold), showing a near 2-fold increase at the end of this period in relation to the initial values. During EC, APX activity decreased 2-fold and then remained unchanged at DR, reaching the initial values (Figure 7a).

During the second season, APX activity declined about 40% at PD in the TA, and peaked at the end of ED, showing a 3.7-fold increase. Then, the activity decreased during EC, returning at the initial values at the end of this period. Finally, APX activity displayed a 65% decrease in relation to the initial values (Figure 7b). In the CA, APX activity remained unchanged at PD, and then an increase was produced at end of ED (1.9-fold) and at the end of EC (2.6-fold). At DR, a near 6-times decline in APX, in relation to initial values, was observed (Figure 7b).

During the first season, in the TA, MDHAR activity increased at the end of PD and at the initial ED, followed by a 40% decrease at the end of ED. MDHAR activity continued declining at the end of EC (2-fold), but showed a 24% increase at DR (Figure 8a). In the CA, MDHAR remained unchanged practically the entire dormancy period. The only significant change was observed at the end of EC, when MDHAR showed a 2.8-fold decline (Figure 8a). During the second season, in the TA, MDHAR activity increased at PD, followed by a decrease at ED and then the activity peak (2-fold the initial values) at the onset of EC. Finally, MDHAR activity returned to the initial values at DR (Figure 8b). In the CA, MDHAR activity maintained constant values at PD, followed by an increase at ED in relation to the values observed at the end of PD. At EC, MDHAR showed no significant changes in relation to the initial values and, finally, a 38% decrease occurred at DR (Figure 8b).

Regarding the DHAR activity, during the first season and in both locations an over-30% increase at the end of ED was followed by an activity peak at the end of EC. Then, the activity at DR reached the initial values in the TA, whereas it dropped by half the initial values in the CA (Figure 9a). During the second season and in both locations, DHAR activity declined at the end of PD and at the onset of EC. Then, DHAR activity peaked at the end of ED in the CA, whereas a strong decrease occurred in both locations at the onset of EC and at DR (Figure 9b).

During the first season and in the TA, the GR activity showed a decline at the end of PD, as well as during ED and at the onset of EC. At the end of EC and at DR, the activity levels recovered but did not reach the initial values (Figure 10a). In the CA, GR decreased at the end of PD and the beginning of ED and EC. However, GR activity reached initial levels at the end of ED and EC as well as at DR (Figure 10a). During the second season, in the TA, GR activity peaked at the end of PD and the initial EC, whereas no significant differences were observed in the other periods with respect to the initial values (Figure 10b). In the CA, GR activity remained statistically unchanged during PD, ED and the onset of EC. Finally, a decline of about 33% was produced at the end of EC and at DR (Figure 10b).

**Figure 12:**
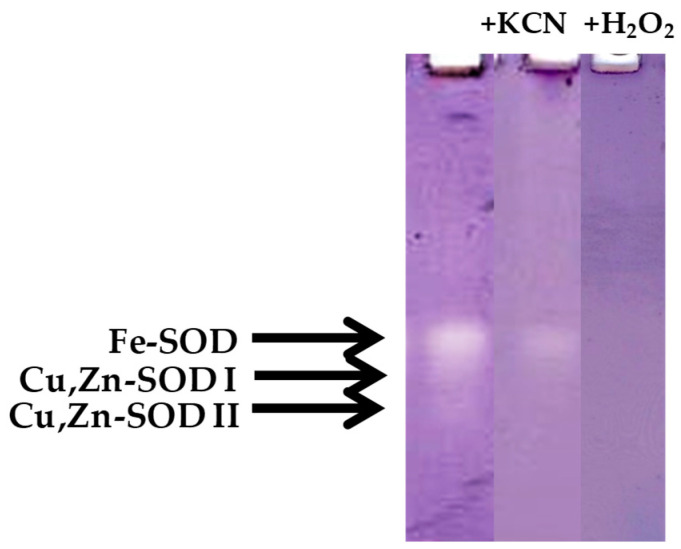
Pattern of superoxide dismutase (SOD) isoenzymes in native PAGE for peach ‘GEM020′ flower buds cultivated in two different geographical areas during the dormancy cycle. Samples from the temperate area at dormancy release are taken as representative. Gels were stained in the presence and in the absence of the selective inhibitors KCN or H_2_O_2_. Ten micrograms of proteins per line were loaded.

Regarding SOD activity, during the first season and in the TA, increased values with respect to the initial ones at ED (250%) and the end of EC (100%) were observed (Figure 11a). Similarly, in the CA, SOD activity peaked at ED (ca. 250%) and then dropped, reaching values below the initial ones at DR (Figure 11a). During the second season and in the TA, SOD activity declined about 25% in ED, and mainly at the end of EC, when a 2.5-fold decrease was observed. Then, SOD activity showed an increase at DR, in relation to the previous phase, but the activity value was still 28% lower than the initial activity (Figure 11b). In the CA, SOD activity declined substantially at PD and ED as well as at the end of EC and at DR. However, SOD activity recovered to the initial values at the onset of EC (Figure 11b). Native PAGE revealed the presence of three different SOD isoenzymes in floral buds from the peach variety GEM020. Figure 12 shows the referred three bands in samples of the TA at DR as representative. According to the results obtained with the selective inhibitors (KCN and H_2_O_2_), a Fe-containing SOD and two Cu,Zn-containing SODs, named I and II in order of increasing mobility, were observed (Figure 12).

**Figure 13:**
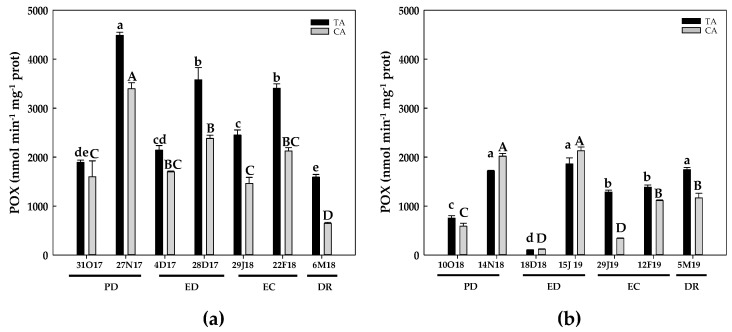
Evolution of peroxidase (POX) activity in flower buds of peach ‘GEM020’ cultivated in two different geographical areas during the dormancy cycle on two seasons. (**a**): season 1 (2017–2018); (**b**): season 2 (2018–2019). Data represent the mean ± SE, *n* = 5. Different letters (lowercase and uppercase for the samples of the temperate (TA) and the cold area (CA), respectively) indicate significant differences according to Tukey’s multiple test (*p* ≤ 0.05). PD: paradormancy; ED: endodormancy; EC: ecodormancy; DR: dormancy release. FW: fresh weight. The chill units accumulated at the different times and locations are shown in Supplemental file 1.

**Figure 14:**
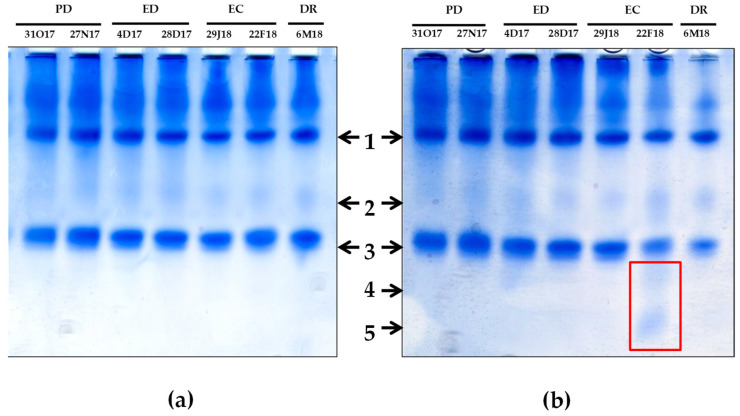
Pattern of peroxidase (POX) isoenzymes in native PAGE for peach ‘GEM020’ flower buds cultivated in two different geographical areas during the dormancy cycle. (**a**)Temperate area; (**b**) Cold area. Numbers (1–5) and arrows indicate bands in increasing order of mobility. Bands 4 and 5, detected only in the cold area, are framed in red. Ten micrograms of proteins per line were loaded.

In the TA and during the first season, POX activity showed a 2.3-fold increase at the end of PD. POX activity also increased near 90% at the end of ED and the end of EC, reaching the initial values at DR. In the CA, POX activity also increased at the end of PD and at the end of ED, whereas a 2.5 decrease occurred at DR (Figure 13a). In the second season, in the TA, POX activity strongly raised at the end of PD, and the activity levels remained higher than the initial ones for the rest of phases except at the onset of ED, when a significant decrease was found (Figure 13b). In the CA, POX activity behaved similar to that observed in the TA, showing an increase at the end of ED, followed by significant declines at the onset of both ED and EC phases with respect to the initial values (Figure 13b). By native PAGE and POX staining, three main bands with POX activity were persistently detected in floral buds during the whole dormancy period for both locations (data not shown). Remarkably, prior to DR and only in the CA, two novel POX isoenzymes were detected in addition to the three abovementioned bands, showing a lower intensity and a higher mobility (Figure 14).

**Figure 15:**
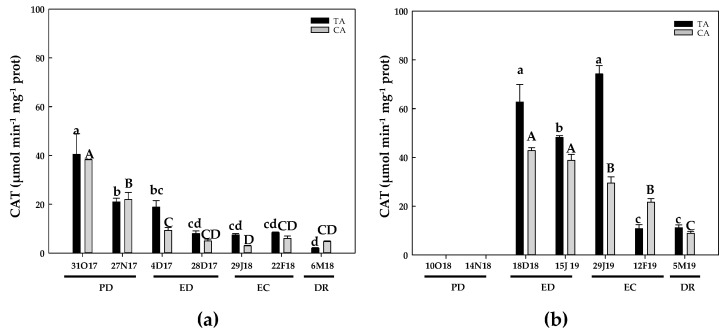
Evolution of catalase (CAT) activity in flower buds of peach ‘GEM020’ cultivated in two different geographical areas during the dormancy cycle on two seasons. (**a**): season 1 (2017–2018); (**b**): season 2 (2018–2019). Data represent the mean ± SE, *n* = 5. Different letters (lowercase and uppercase for the samples of the temperate (TA) and the cold area (CA), respectively) indicate significant differences according to Tukey’s multiple test (*p* ≤ 0.05). PD: paradormancy; ED: endodormancy; EC: ecodormancy; DR: dormancy release. FW: fresh weight. The chill units accumulated at the different times and locations are shown in Supplemental file 1.

During the first season, the CAT activity displayed a progressive decrease during the dormancy period in both locations, reaching a minimum at DR, mainly in the TA (Figure 15a). In the second season, CAT activity was not detected at PD. Then CAT activity was detected at ED and, as in the first season, declined at the end of EC and DR compared to the initial values (Figure 15b).

### 3.4. Hormone Metabolism

The levels of bioactive GA (GA_1_, GA_4_ and GA_7_) were determined in samples from the two growing seasons, GA_7_ being the most abundant in both areas.

**Figure 16:**
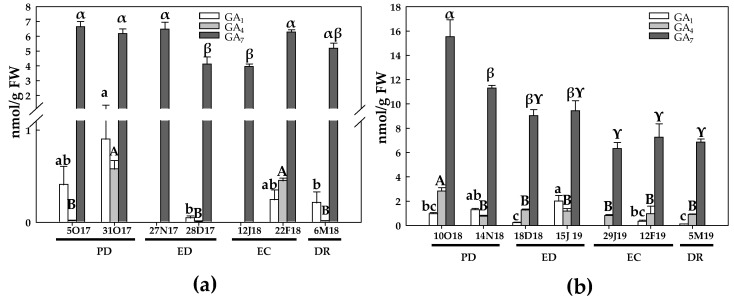
Evolution of gibberellins contents in flower buds of peach ‘GEM020’ cultivated in a temperate area during the dormancy cycle on two seasons. (**a**): season 1 (2017–2018); (**b**): season 2 (2018–2019). Data represent the mean ± SE, *n* = 5. Different letters (lowercase, uppercase and Greek letters for GA_1_, GA_4_ and GA_7_, respectively) indicate significant differences according to Tukey’s multiple test (*p* ≤ 0.05). PD: paradormancy; ED: endodormancy; EC: ecodormancy; DR: dormancy release. FW: fresh weight. The chill units accumulated at the different times and locations are shown in Supplemental file 1.

During the first season and in the TA, GA_1_ levels remained unchanged during PD. However, at the end of ED, a 9-fold decrease on GA_1_ levels took place compared to the initial values. In addition, this GA was not detected at the onset of both ED and EC. On the other hand, no significant changes were observed at EC and at DR periods (Figure 16a). The initial GA_4_ levels were very low, as occurred at the onset of ED and at DR, whereas this hormone was not detected at the beginning of both ED and EC. In contrast, GA_4_ concentration was strongly induced at the end of PD (32-fold) and at the end of EC (22-fold), and then declined at DR (Figure 16a). The GA_7_ levels were unchanged at PD, but decreased about 40% at ED. However, GA_7_ levels increased again at EC and at DR (Figure 16a). During the second season, GA_1_ levels were unchanged during the PD in the TA. Then, GA_1_ levels decreased at the onset of ED and subsequently increased at the end of ED (2-fold). Finally, GA_1_ levels decreased at the end of EC as well as at DR, although the differences were not significant in relation to the initial values. The GA_4_ contents experienced a significant decrease during the dormancy period, in relation to the initial values, showing a decline ranging from 2.2 to 3.7-fold during the mentioned period (Figure 16b). Finally, GA_7_ levels progressively declined during the dormancy period, reaching the lowest values at EC and at DR (Figure 16b).

**Figure 17:**
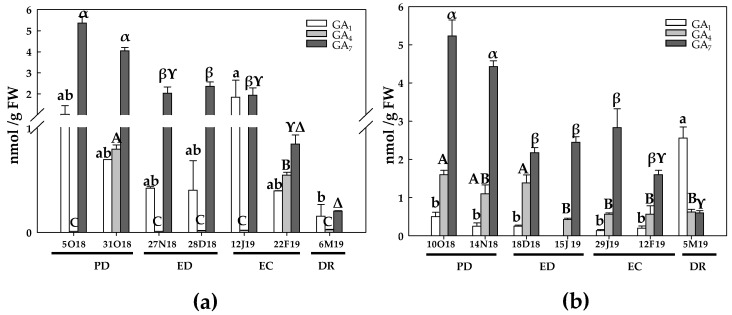
Evolution of gibberellins contents in flower buds of peach ‘GEM020’ cultivated in a cold area during the dormancy cycle on two seasons. (**a**): season 1 (2017–2018); (**b**): season 2 (2018–2019). Data represent the mean ± SE, *n* = 5. Different letters (lowercase, uppercase and Greek letters for GA_1_, GA_4_ and GA_7_, respectively) indicate significant differences according to Tukey’s multiple test (*p* ≤ 0.05). PD: paradormancy; ED: endodormancy; EC: ecodormancy; DR: dormancy release. FW: fresh weight. The chill units accumulated at the different times and locations are shown in Supplemental file 1.

During the first season and in the CA, the GA_1_ contents were statistically unchanged, its maximum value being recorded at the onset of EC and the lowest at DR (Figure 17a). The initial GA_4_ levels were very low, as observed at ED. In this case, a dramatic increase in GA_4_ occurred at the end of PD and EC (Figure 17a). GA_7_ contents gradually decreased from PD to DR near 90% (Figure 17a). During the second season, GA_1_ levels were unchanged during the dormancy process, but a 20-fold increase took place at DR. GA_4_ levels were constant during PD and at the onset of ED. However, from that moment on, GA_4_ levels showed a decrease (3 to 4-fold) until DR (Figure 17b). GA_7_ levels, similarly to that observed in the first season, reached the lowest value at DR (about a 9-fold decrease) (Figure 17b).

**Figure 18:**
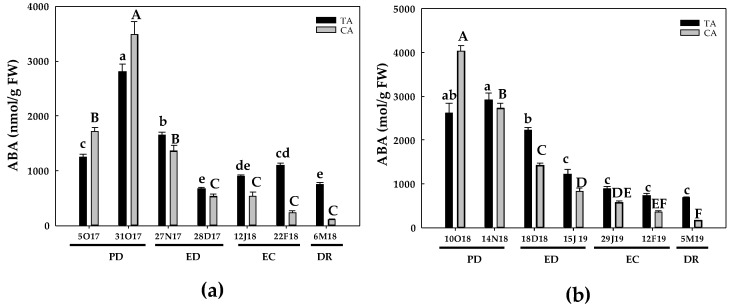
Evolution of abscisic acid (ABA) content in flower buds of peach ‘GEM020’ cultivated in a cold area during the dormancy cycle on two seasons. (**a**): season 1 (2017–2018); (**b**): season 2 (2018–2019). Data represent the mean ± SE, *n* = 5. Different letters (lowercase and uppercase for the samples of the temperate (TA) and the cold area (CA), respectively) indicate significant differences according to Tukey’s multiple test (*p* ≤ 0.05). PD: paradormancy; ED: endodormancy; EC: ecodormancy; DR: dormancy release. FW: fresh weight. The chill units accumulated at the different times and locations are shown in Supplemental file 1.

**Figure 19:**
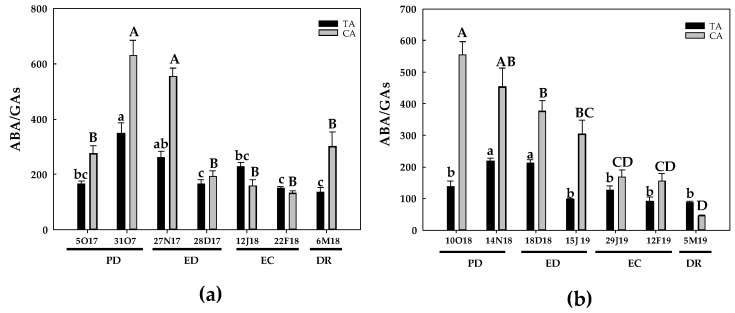
Evolution of abscisic acid/total gibberellins (ABA/total GAs) in flower buds of peach ‘GEM020’ cultivated in a cold area during the dormancy cycle on two seasons. (**a**): season 1 (2017–2018); (**b**): season 2 (2018–2019). Data represent the mean ± SE, *n* = 5. Different letters (lowercase and uppercase for the samples of the temperate (TA) and the cold area (CA), respectively) indicate significant differences according to Tukey’s multiple test (*p* ≤ 0.05). PD: paradormancy; ED: endodormancy; EC: ecodormancy; DR: dormancy release. FW: fresh weight. The chill units accumulated at the different times and locations are shown in Supplemental file 1.

Regarding ABA content, during the first season and in both locations, a peak was observed at PD, followed by a progressive decline until DR. However, such decrease was more pronounced in the CA. Therein, ABA levels were 15-times lower than the initial levels, whereas in the TA, the ABA decline was about 40%, in relation to its initial contents (Figure 18a). During the second season, the behavior of ABA content during the dormancy period was similar, with a progressive decrease in ABA content during the dormancy period. Again, the decrease in ABA content was more pronounced in the CA than in the TA. In this case, a 4-fold decrease in ABA was noticed in the temperate zone at DR, whereas a near 9-fold decline was observed in the CA at the same phase (Figure 18b). As a consequence of the decline in ABA content, a reduction in the ABA/total GAs ratio was observed in both seasons and areas, although this response was more evident in the CA, especially during the second season (Figure 19).

Principal components analysis (PCA) was used as a mathematical tool to determine associations among the different variables studied. Four PCA models were elaborated, resulting from the data analysis of each area/season combination (Supplemental file 2). PCA resulted in models with two principal components (PCs) explaining about 64% of the total variance in the TA (Supplemental files 2a,b) and about 60% of the total variance in the CA (Supplemental files 2c,d). In the TA, only starch, DHAR, and MDHAR showed similar loadings distribution between both seasons, indicating that these parameters are important factors involved in DR, being the rest of variables tested likely more influenced by the climatic characteristics of each season. This could rely on physiological disorders produced by deficient chilling accumulation in the TA. However, in the CA, in which the peach variety fulfills chilling requirements, three groups of variables [(starch, GR and DHAR), (fructose and glucose), (sucrose and APX)] showed similar loadings distribution between both seasons. Taken together, these results highlight the importance of sugar metabolism and ascorbate recycling during bud dormancy and bud burst in the CA.

## 4. Discussion

The present work shed light on the physiological and biochemical mechanisms involved in bud dormancy and release, which may contribute to devising agronomic strategies for managing bud burst and therefore harvest. Moreover, this knowledge would help breeders in the selection of adequate cultivars depending of the climatic characteristics of the area of interest. This is particularly interesting in crops with high yields and short harvest periods like peach.

### 4.1. Histological Analysis

Although the rate of the pollen grain development is genetically determined, the effect of the climate conditions on this process is critical [33]. In the present work, we have observed that the lack of chill in the TA delayed and could even compromise pollen development. In the CA, a number of pollen grains appeared in the anthers of the high chilling peach GEM020 growing in the CA in March. However, at this moment, the development of the anthers was interrupted in the TA, where pollen mother cells still appeared. The increased starch content observed at EC (12th February) in both areas, correlated with the development of the pollen mother cells. The more advanced developmental stage of the anthers observed in the CA could also be related with the starch levels. Moreover, some microspores were observed in the CA in the middle of February, before the estimated end of the dormancy period. According to this, GEM020 cultivated in the CA could have its chilling requirements satisfied by the middle of February (Figure 1).

Different authors indicated that the appearance of pollen tetrads and the beginning of microsporogenesis can be considered the end of dormancy in apricot [34,35] and peach [36]. The interruption of anthers development observed in the TA can be related to the climate conditions in this area. It is well known that this peach variety does not fulfil chilling requirements in the TA. Moreover, it is known that when 60–70% of the cold requirements have been satisfied, if there is a rise in temperatures afterwards, this variety can break dormancy but vegetative disorders occur. In this work, this type of disorders would be manifested in the TA by the interruption of anthers development. In addition, insufficient chilling accumulation can have undesirable effects, such as deficient flowering, lack of fruit set or unequal foliation [29].

### 4.2. Carbohydrate Metabolism

Reserve carbohydrates act as the primary source of carbon and energy in the process of bud DR [37]. In addition, sugars may act as signaling molecules in plant development regulation [11,38]. Chilling temperatures affect carbohydrate metabolism in the subjacent tissues of flower buds. Thus, C and N reserves in the bark and xylem tissues of twigs are mobilized to support the growth restart of the vegetative and floral buds [14]. In stem cutting of four different *Prunus* species, artificial high chilling treatments (up to 1000 chilling hours, CH) decreased starch contents in the bark tissue, whereas glucose concentration significantly increased in the bark tissue by the effect of up to 500 CH [14]. Additional chilling, i.e., 1000 CH, decreased glucose contents only in plum, but did not alter its levels in peach, apricot and nectarine. An analogous behavior occurred with fructose, but only in apricot [14]. Sorbitol contents also decreased with the chilling treatment in peach, nectarine and plum [14]. Overall, our results are in agreement with these observations, even though different experimental model and tissue were used. The effect of low temperature on starch and sugars contents during the rest of the period could rely on induced α-amylase activity by cold temperature, which would increase starch hydrolysis and consequently sugar contents, as described in peach [39].

In this work, sucrose and sorbitol were the major sugars in peach buds from the GEM020 variety (Figure 3 and Figure 5), as previously described in a low chill peach variety (GEM065) [26]. Although the starch contents evolved differently during dormancy process for the two studied periods (Figure 2), a clear decrease in starch content from EC to DR was observed in both years and locations. This correlated with an increase in glucose (Figure 6) and fructose levels, mainly during the first season, which in turn would support the flower differentiation. The decrease in starch at DR was also reported in lateral buds from two walnut cultivars, differing in chilling requirements [15] as well as in flower buds from a low-chill peach variety [26]. In parallel, in walnut buds, these authors also observed a progressive increase in total soluble sugars, glucose and fructose, whereas a biphasic response for sucrose occurred, i.e., a progressive increase followed by a decrease previous to DR [15]. This is in agreement with our results in peach buds, where sucrose levels decreased from ED to DR, the response being more evident in the first studied period (2017–2018).

Increased soluble sugars may improve bud tolerance to low temperatures by decreasing the freezing point of free water, avoiding the formation of ice crystals. In addition, sugars can also supply C skeleton for the primary metabolism in relation to bud growth, development and differentiation [12]. In that regards, an increase in soluble sugars, sucrose and glucose has also been reported in buds of *Prunus mume* at bud burst [12]. Therefore, the increase in glucose and fructose can improve the response of peach buds to low temperatures, especially in the CA.

### 4.3. Antioxidant Metabolism

In peach GEM020 floral buds all the ASC-GSH cycle enzymes as well SOD, POX and CAT activities were detected (Figure 7, Figure 8, Figure 9, Figure 10 and Figure 11). The time-course of the different enzyme activities pattern did not resemble for both studied seasons. However, some common patterns for some specific enzymes were observed. In this sense, a decline in APX, DHAR, SOD and CAT activities occurred in both seasons at DR, suggesting that a tight control of H_2_O_2_ and O_2_^•−^ accumulation may take place during DR. This progressive decrease in APX activity also occurred in floral pear buds at the late-breaking period (when a 96% of bud break occurred), followed by a sharp increase of the activity [40]. However, in walnut buds, APX progressively increased during the dormancy period, peaking at DR [15]. In a low-chill peach variety, a decrease in APX, MDHAR and CAT activity was also reported in floral buds at DR [26]. This suggests that increased ROS content mediated by decreased antioxidant capacity could be a common mechanism for controlling the transition from ED to EC in peach and other stone fruits. In addition, the drop in DHAR activity also at DR could lead to a more oxidized environment by compromised ascorbate recycling.

ROS have been involved in stress-induced flowering [18,41,42] and in modulating gene expression [43]. In this sense, different evidences suggested a role of ROS in DR. For example, diphenylene iodonium chloride, a NADPH oxidase inhibitor, promoted potato tuber sprouting via increased ROS accumulation [44]. On the other hand, the application of hydrogen cyanamide, a catalase inhibitor, provoked an increased ROS-mediated floral bud break in grapevine [19,22] and sweet cherry [5]. This accumulation was linked to the decrease in catalase activity [5,45,46] and the increased expression of oxidative stress-related transcripts [5].

Remarkably, flower buds of peach GEM020 contained a Fe-SOD isoenzyme but not a Mn-SOD isoenzyme (Figure 12). This may be related to the H_2_O_2_-sensitivity of Fe-SOD and Cu,Zn-SOD isoenzymes, and the H_2_O_2_-resistance of Mn-SOD [47].This SOD isozyme profile in floral buds was previously found in the peach variety GEM065, characterized by low chill requirements [26]. Fe-SOD has been located in chloroplasts, whereas Mn-SOD has a mitochondrial and peroxisomal location [48]. However, Fe-SOD has also been found in mitochondria and peroxisomes from carnation flowers [49].

In addition, we observed the up-regulation of two minor POX isoenzymes previous to DR, but only in the CA. This result suggests a possible role of both POX isoenzymes in the DR process and their use as markers for monitoring DR in high-chill peach varieties grown in cold areas. In a similar way, in a low-chill peach variety, a novel POX isoenzyme was induced prior to DR and, with more intensity, during DR [26]. As in the present work, this new POX isoenzyme also showed lower intensity and higher mobility than the main POX bands observed in the native gel. Moreover, our results agree with a recent work in almond buds from three different chilling-requirements cultivars [50], where an increased expression of Class III peroxidase gene (named *PdP40*) was associated to enhanced POX activity before ED release [50]. Ref. [10] also reported the induction of different peroxidase-like genes in peach buds at DR stage. Taken together, a role for these induced POXs as well as for H_2_O_2_ in the process of bud-breaking regulation can be suggested.

### 4.4. Plant Hormones

Plant hormones have a key role in both the induction and the breaking of bud dormancy [4]. However, most of the research in this regard has been conducted in Arabidopsis [13], the studies on *Prunus* species being more scarce. Seed dormancy resembles bud dormancy regarding the hormonal response: ABA promotes the establishment of seed dormancy during the seed development [51]. In this sense, a similar role for ABA during bud formation and dormancy has been reported [1]. It has been widely accepted that endogenous ABA content increase at dormancy establishment and decrease towards DR, which is concomitant with chilling accumulation [52]. For example, in *Prunus mume*, ABA contents were high at the beginning of ED, and progressively decreased until DR [12]. Similar results were reported in peach [53] and in some other woody species such as pear [52,54] and sweet cherry [55]. Some works determined that ABA is only important for bud establishment and maintenance, but not for DR [5]. During EC of floral buds from peach and apricot, ref. [24] described the up-regulation of 3 genes involved in ABA metabolism and 15 genes responsible for ABA stimulus. Among them, genes involved in the inhibition of ABA accumulation and the down-regulation of ABA signaling were found [24]. We observed a high ABA concentration at PD in GEM020, and a similar result was also evident in the peach variety GEM065 [26], although the ABA levels were much higher in the high-chill variety (GEM020) than in the low-chill variety (GEM065).

GA and ABA are considered to have antagonistic effects in the control of seed germination and dormancy of flower buds [12,51]. However, the role of GA on bud burst is not clear, and both promoter and inhibitory effects have been described, influenced by several factors such as the developmental stage of the buds and the type of GA [5,56,57]. In peach and apricot floral buds, the expression of two *GA2-oxidase* genes, involved in GA deactivation, were down-regulated at ED, favoring the increase in the bioactive GA levels on the transition from ED to EC and the reactivation of plant growth [24]. However, the expression of *GA20-oxidase* genes, involved in GA biosynthesis, was unchanged [24]. These results suggest that suppression of GA deactivation is more important than GA biosynthesis for the DR process [24]. This is in agreement with that observed by [12], who showed a sharply increase in GA_3_, GA_1_ and GA_4_ at DR in *Prunus mume* floral buds. As a result of the increase in GA contents and the decline in ABA contents, Zhang et al. (2018) recorded a progressive decrease in the ABA/GA ratio during the dormancy period in *Prunus mume* [12]. These results contrast with our study, in which the levels of GA_7_, the main bioactive GA, declined at DR, as also occurred in the low-chill peach variety GEM065 [26]. In the present work, a strong decrease in ABA/GA ratio during the progression of the dormancy was observed, reaching its minimum level at DR. However, this decline was mainly due to the decline in ABA rather than to an increase in GAs, as described by other authors. Similar results in ABA/GAs ratio were also reported in grapes and sweet cherry [58,59]. Thus, it seems that the ABA/GA balance is more important for the dormancy process than the endogenous levels of ABA and GAs [12,26]. In our case, we can assume that GAs are not important for bud burst in any geographical location studied. Although other plant hormones that may be involved in DR have not been analyzed, we can suggest that a decline in ABA and hence in ABA/GA ratio is crucial for bud burst in peach.

In addition, a cross-talk between sugars and plant hormones during the flowering induction has been reported [12,13]. In Arabidospis, it has been described a GA- and ABA-dependent regulation of α-amylases, as well as an influence of sugars in hormone metabolism through an UDP-glycosilation mechanism, leading to inactive forms of hormones [60]. In our work, to establish an interplay between hormone and sugar pathways, future in depth studies involving signaling elements and transcription analyses are needed.

**Figure 20:**
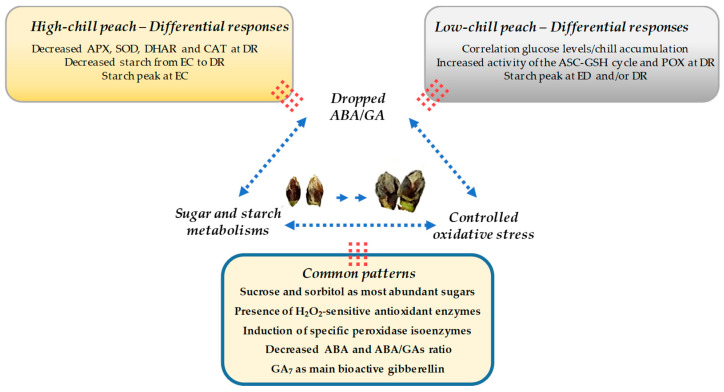
Comparative scheme of the physiological events occurring in peach buds during dormancy evolution and release. The differential responses and the common patterns for a high-chill (this study) and a low-chill peach variety [26] are shown. In synthesis, an interplay among a controlled oxidative stress, changes in sugar and starch metabolisms, and decreased ABA/GAs ratio is proposed to drive dormancy breaking. ASC-GSH: ascorbate-glutathione; APX: ascorbate peroxidase; CAT: catalase; DHAR: dehydroascorbate reductase; DR: dormancy release; EC: ecodormancy; ED: endodormancy; POX: peroxidase; SOD: superoxide dismutase.

Finally, by establishing a comparison between the results of this study and the obtained recently in our research group on a low-chill peach cultivar [26], common determinants of peach bud dormancy maintenance and release can be suggested for both high- and low-chill peach (Figure 20). In both cases, the presence of H_2_O_2_-sensitive antioxidant enzymes in the buds suggests a controlled oxidative stress leading to DR and remarks the importance of the antioxidant metabolism in this process. Moreover, the novel POX isoenzyme observed at DR could be used as a marker for this event in low- and high-chill peach varieties grown in different climatic zones. In addition, a progressive decrease of ABA/GAs ratio was a common pattern (Figure 20). In contrast, the noticeable differences on the evolution of the main sugars between both types of cultivars suggests that sugar metabolism and related signaling events can be more cultivar-specific and/or associated with the chill requirements.

## 5. Conclusions

In the present work, a correlation among fructose and glucose accumulation, decreased starch content, and reduced ABA content and ABA/GAs ratio was observed at DR. In addition, sucrose and sorbitol were the major sugars in peach buds from the GEM020 variety. A decline in APX, SOD, DHAR and CAT activities in both season of study was observed at DR, suggesting that sub-lethal H_2_O_2_ and O_2_^•−^ accumulation may lead to an oxidative signaling required for DR and subsequent development and differentiation of peach buds [17]. According with this hypothesis, peach floral buds contain the H_2_O_2_-sensitive Fe-SOD and Cu,Zn-SOD isoenzymes but not the H_2_O_2_-resistant Mn-SOD. The presence of H_2_O_2_-sensitive antioxidant enzymes found in peach buds could cause the establishment of a controlled oxidative stress that may be related to signaling events governing the DR process. In addition, the induction of two new POX isoenzymes previous to the DR suggests a possible role of certain POX isoenzymes and hence for H_2_O_2_, in the DR process. Thus, if characterized, these POX isoenzymes could be used as markers of DR in peach.

PCA showed different parameters that behave in a similar way in the two studied seasons. This was more evident in the CA, in which carbohydrates and the enzymes DHAR and GR were grouped in the same way in both seasons. Furthermore, in the TA, starch content and ascorbate recycling activities were also distributed in the same way in the two studied seasons. This suggest that in all cases, sugar metabolism as well as the recycling of ascorbic acid, could play an important role in the dormancy process in peach buds.

In synthesis, a possible interaction sugars-hormones-ROS could take place in high-chill peach buds, favoring the DR process, suggesting that redox interactions as well as sugar metabolism can govern bud DR, regardless of chilling requirements.

## Data Availability

Data Availability Statements in section “MDPI Research Data Policies” at https://www.mdpi.com/ethics (accessed on 24 February 2021).

## Supplementary Materials

The following are available online at https://www.mdpi.com/article/10.3390/antiox10040560/s1, Supplemental file 1: Dates for flower bud sampling and corresponding dormancy stages and chill units accumulated for the two experimental growing seasons and locations. Supplemental file 2: Principal component analysis (PCA) applied to sugars content, starch content, antioxidant enzyme activities and hormone levels in flower buds of peach ‘GEM020’ cultivated in two different geographical areas during the dormancy cycle on two seasons.
