# Supplementary material for: Interplay among Antioxidant System, Hormone Profile and Carbohydrate Metabolism during Bud Dormancy Breaking in a High-Chill Peach Variety
Source: Antioxidants (Basel). 2021 Apr 4;10(4):560. doi: 10.3390/antiox10040560 (PMC8066612; doi:10.3390/antiox10040560)
Supplement: Supplementary file 1 [file antioxidants-10-00560-s001.pdf]

Supplemental File S1. Dates for flower bud sampling and corresponding dormancy stages and chill portions accumulated for the two experimental growing seasons and locations.

2017-2018	Paradormancy			Endodormancy			Ecodormancy			Dormancy release
**Date**	5/10/17	31/10/17	27/11/17	4/12/17	28/12/17	12/1/18	29/1/18	12/2/18	22/2/18	6/3/18
**Chill portions TA**	0	0	11	15.7	30.1	42.4	46.6	56.2	63	66.8
**Chill portions CA**	0	2	13.5	18	32.5	41.6	53.4	62.5	69.2	76.8

2018-2019	Paradormancy		Endodormancy		Ecodormancy		Dormancy release
**Date**	10/10/ 18	14/11/18	18/12/18	15/1/19	29/1/19	12/2/19	5/3/19
**Chill portions TA**	0	0	22.8	41.8	50.7	58.4	69.8
**Chill portions CA**	0	13.2	34	50.7	59.7	68.2	79.6

TA: temperate area; CA: cold area.

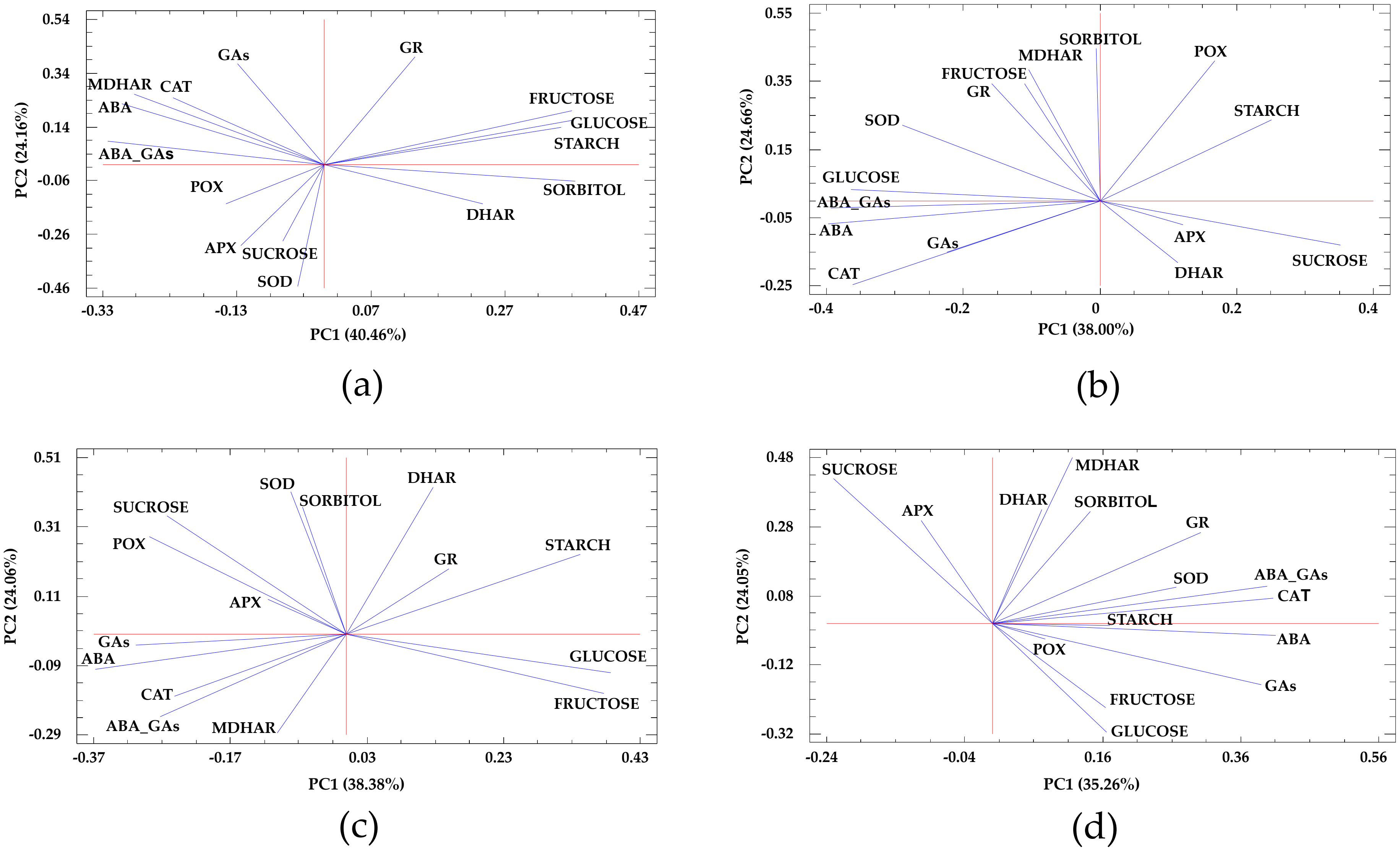

(a)
PC1 (40.46%)
PC2 (24.16%)
0.54
0.34
0.14
-0.06
-0.26
-0.46
-0.33
-0.13
0.07
0.27
0.47
GAs
GR
MDHAR
CAT
ABA
FRUCTOSE
GLUCOSE
STARCH
ABA_GAs
POX
SORBITOL
DHAR
APX
SUCROSE
SOD
(b)
PC1 (38.00%)
PC2 (24.66%)
0.55
0.35
0.15
-0.05
-0.25
-0.4
-0.2
0
0.2
0.4
SORBITOL
MDHAR
POX
FRUCTOSE
GR
STARCH
SOD
GLUCOSE
ABA_GAs
ABA
APX
GAs
SUCROSE
CAT
DHAR
(c)
PC1 (38.38%)
PC2 (24.06%)
0.51
0.31
0.11
-0.09
-0.29
-0.37
-0.17
0.03
0.23
0.43
SOD
DHAR
SORBITOL
SUCROSE
POX
GR
STARCH
APX
GAs
ABA
GLUCOSE
CAT
FRUCTOSE
ABA_GAs
MDHAR
(d)
PC1 (35.26%)
PC2 (24.05%)
0.48
0.28
0.08
-0.12
-0.32
-0.24
-0.04
0.16
0.36
0.56
SUCROSE
MDHAR
DHAR
APX
SORBITOL
GR
SOD
ABA_GAs
CAT
STARCH
ABA
POX
GAs
FRUCTOSE
GLUCOSE

SupplementaL File S2. Principal component analysis (PCA) applied to sugars content, starch content, antioxidant enzyme activities and hormone levels in flower buds of peach 'GEM 020' cultivated in two different geographical areas during the dormancy cycle on two seasons. (**a**): PCA for season 1 (2017-2018) in the temperate area (TA); (**b**): PCA for season 2 (2018-2019) in the TA; (**c**): PCA for season 1 in the cold area (CA); (**d**) PCA for season 2 in the CA. The blue lines denote eigen vectors characterised by the direction and the strength of the variable relative to principal component (PC) 1 and 2. ABA: abscisic acid; APX: ascorbate peroxidase; CAT: catalase; DHAR: dehydroascorbate reductase; GAs: total gibberellins; MDHAR: monodehydroascorbate reductase; POX: peroxidase; SOD; superoxide dismutase.
